# Unilateral Biportal Endoscopic Laminectomy-Bilateral Decompression Using a Monoportal Scope and Bipolar Coagulator for Lumbar Spinal Stenosis: A Technical Report

**DOI:** 10.7759/cureus.46944

**Published:** 2023-10-13

**Authors:** Takeshi Kaneko, Yuichi Takano, Hiroki Iwai

**Affiliations:** 1 Spine Surgery, Inanami Spine and Joint Hospital, Tokyo, JPN; 2 Spine Surgery, Iwai Orthopaedic Hospital, Tokyo, JPN

**Keywords:** radio-frequency ablation, lumbar spinal decompression, lumbar spinal stenosis (lss), full endoscopic spine discectomy, unilateral biportal endoscopic surgery

## Abstract

The purpose of this study was to introduce the application of a monoportal scope and bipolar coagulator used in full-endoscopic spine surgery (FESS) for unilateral biportal endoscopy-unilateral laminectomy bilateral decompression (UBE-ULBD) in those with central stenosis. A 68-year-old man who presented with cauda equina symptoms underwent UBE-ULBD to improve his central stenosis at the L2/3 level. In this technique, a FESS scope was attached to a camera portal in place of a common arthroscope. A decompression tool was subsequently inserted through the working portal, and the lower border of the vertebral lamina and the lower border of the contralateral lamina were resected. Additionally, the superior border of the L3 level was thinned using a high-speed drill, and the ligament flavum was excised. The operation time was 70 minutes, and his symptoms improved. The patient was discharged from the hospital four days postoperatively. We found three advantages of using a FESS scope and bipolar coagulator, including the ability to 1) stabilize the camera via placement of the sleeve against the bone, 2) minimize the wounded area by irrigating saline on the side of the scope, and 3) provide bipolar tissue hemostasis in an isolated area around the nerves. Therefore, among the UBE techniques, we believe that assisted full-endoscopic spine surgery (AFESS) is a viable option to offer a more minimally invasive surgery for patients with stenosis.

## Introduction

With the rapid development of arthroscopic surgical techniques for patients with stenosis, technical advances have offered a higher degree of freedom due to the use of two independent portals for viewing and working. The improved surgical field and unrestricted working space provide a useful means for decompression surgery.

Unilateral biportal endoscopy (UBE) has been shown to decompress a greater area in the same amount of time compared to conventional micro-endoscopic and full-endoscopic spine surgery (FESS) decompression techniques [1,2]. On the other hand, FESS is a less invasive procedure with an incision of approximately 7 mm, allows the surgical operation to be performed within a protective sleeve, and can be completed with one portal for fluid irrigation [3]. The high frequency (1.7-4.0 MHz) bipolar (HFB) electrocoagulation system used in FESS is a type of bipolar coagulator that energizes the tip alone. Although it is possible to perform decompression in FESS using a drill, one of the drawbacks is the amount of time required to decompress a wide area using a narrow sleeve. Considering the time-consuming decompression procedure in FESS, the creation of an extra working portal will eliminate the spatial limitation of the sleeve and enable the use of a larger resection tool such as the HFB used in FESS. This technical report introduces the application of a monoportal scope and bipolar coagulator used in full-endoscopic spine surgery (FESS) for unilateral biportal endoscopy-unilateral laminectomy bilateral decompression (UBE-ULBD) in a patient with central stenosis.

## Technical report

**Figure 1:**
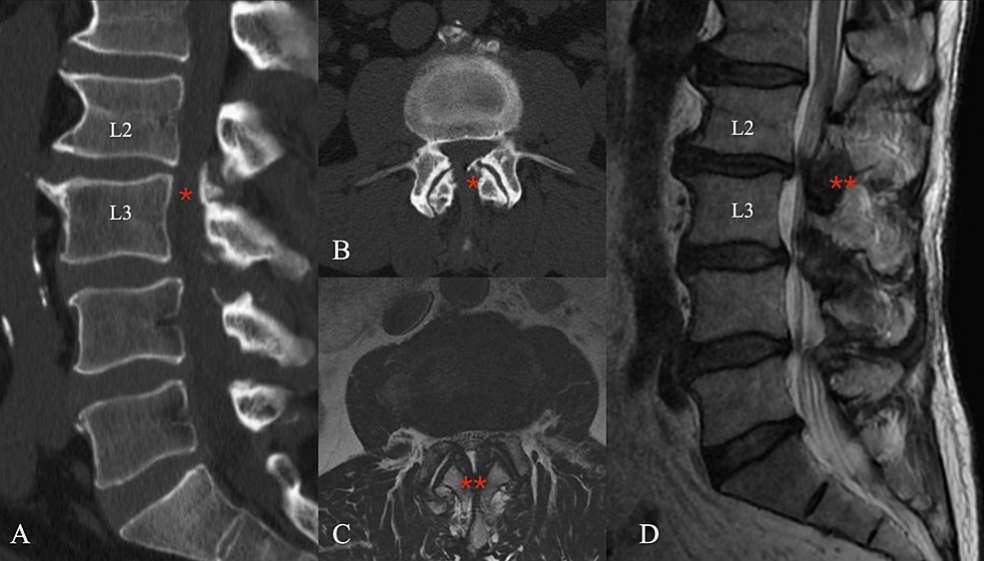
Preoperative imaging (A) Sagittal CT confirmed the protrusion of osteophytes (*) from the superior border of the vertebral arch at the L3 level (B), and osteophytes were confirmed to lie within the spinal canal. (C, D) Axial and sagittal MRI showed that the thickening of the ligamentum flavum (**) was compressing against the spinal canal.

**Figure 2:**
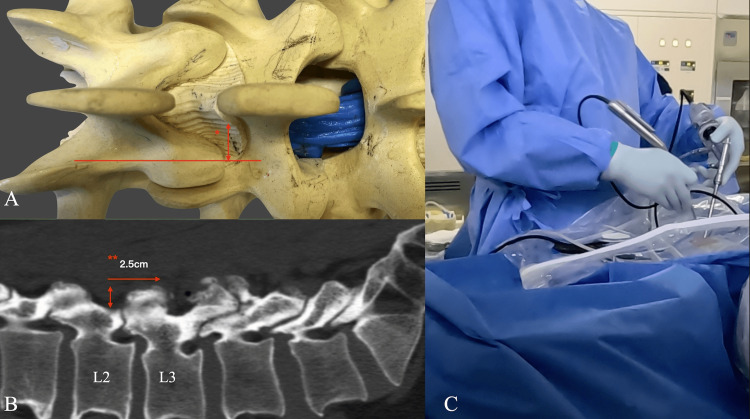
Determining the position of a skin incision (A) Preoperative axial measurements were taken from the spinous process to the inner edge of the superior articular process (SAP) (*). (B) In the lateral fluoroscopic view, the inferior border of the L2 pedicle was determined as the camera portal, and the working portal was placed 2.5 cm caudally from the camera portal (**). (C) Entire operative view during the surgery.

**Figure 3:**
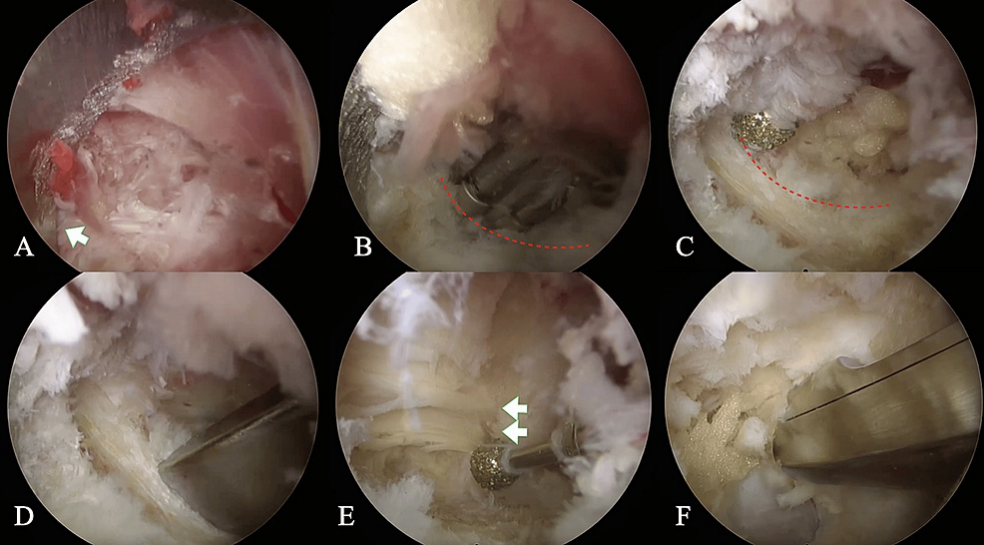
Operative procedure (A) The sleeve was brought into contact with the inferior border of the L2 lamina to stabilize the field of view. (B) The edge of the inferior lamina (dotted line) was clarified with a bipolar coagulator. (C) A drill was used to excise along the dotted line. (D) A chisel was used to enlarge the bony resection. (E) The attachment of the ligamentum flavum at the L3 level (double arrow) was thinned, and the ligament was removed. (F) The ligamentum flavum was resected with a curved Kerrison rongeur on the contralateral side.

**Figure 4:**
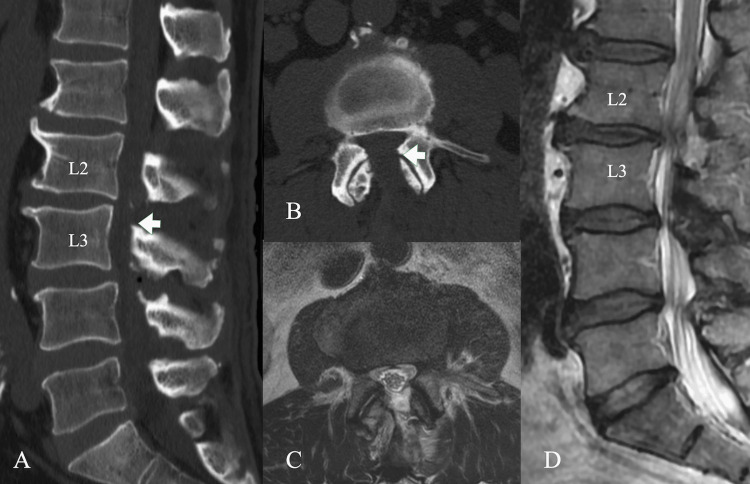
Postoperative imaging (A) Sagittal CT confirmed that the osteophytes were resected (arrow). (B) Axial CT confirmed the osteophytes extending from the superior articular process were resected (arrow), with bony resection on both the entry and contralateral sides. (C) Axial MRI showed that the ligamentum flavum was removed on both sides. (D) Sagittal MRI showed decompression at the L2 and L3 levels.

A 68-year-old man complained of intermittent claudication. MRI revealed central stenosis with thickening of the ligamentum flavum and protrusion of osteophytes at the L2/3 level. (Figures 1A-1C) Because the patient was unable to make progress conservatively with medication and injections, surgery was planned. The patient was placed in the prone position, and surgery was performed under general anesthesia. Preoperative axial CT was used to measure the distance from the spinous process to the inner edge of the superior articular process at the level of the superior border of the L3 vertebral body, where the lateral position of the incision was determined (Figure 2A). Surgery was planned for entry from the left side of the patient. Using a lateral fluoroscopic view, a camera portal was placed at the inferior border of the L2 pedicle, followed by a working portal that was positioned 2.5 cm caudally (Figure 2B). A 7-mm skin incision was created for both portals. A pencil dilator was inserted through the camera portal, wherein the inferior border of the L-2 lamina was confirmed and a beveled sleeve was inserted. Then, a FESS scope (VERTEBRIS, Richard Wolf GmbHR, Knittlingen, Germany) was inserted (Figure 2C). An HFB electrocoagulation system (Trigger-Flex® Quad, Elliquence LLC, Baldwin, NY, USA) was inserted through the working portal, and its tip was placed in a position where it could be visualized. The scope was stabilized by placing the beveled end of the sleeve against the bone (Figure 3A), and the flattened tip of the HFB was used to coagulate the soft tissue while clarifying the bony edges of the lower border on the vertebral arch (Figure 3B). The resection area from the cranial side was decompressed based on the extent to which the ligament flavum was thinned. The contralateral side was subsequently decompressed while leaving the ligamentum flavum intact. The inferior border of the vertebral arch on the contralateral L-4 level was initially excised with a high-speed drill (3.5 mm Primado2 Long, NSK-Nakanishi Japan, Tokyo, Japan) and then with a chisel (Figure 3C). Decompression was performed to the extent that the contralateral superior articular process was visible. Next, the superior border of the L3 lamina was thinned with a drill, and the ligament flavum was removed en bloc (Figures 3D-3F). HFB was used to control the bleeding in the epidural venous plexus, and the required extent of decompression was determined by visualizing the outer surroundings of the dural canal. Finally, when no new bleeding was confirmed, the surgery was completed. A Jackson-Pratt® Hemaduct® Wound Drains was placed for two days. A postoperative CT scan showed that bone fragments had been removed sufficiently (Figures 4A-4B), and the patient was discharged four days later. A two-week postoperative MRI showed that the L3 nerve root had been decompressed (Figures 4C-4D).

## Discussion

A 2002 report by Yeung suggested that a bi-portal approach may be used instead of a single-portal if biportal access is required [4]. After performing surgery for the above case, we discovered three benefits of using a FESS scope and bipolar coagulator, including the ability to 1) stabilize the camera via placement of the sleeve against the bone, 2) minimize the wounded area by irrigating saline on the side of the scope, and 3) provide bipolar tissue hemostasis in an isolated area around the nerves. The details of these three benefits are described below.

Field of view and camera stabilization via placement of the sleeve against the bone

Current commonly used UBE scopes have originated from arthroscopes and have been designed to utilize a fixed sleeve that is the same length as the scope. There is no fluid irrigation area from the side of the scope. Under normal intraarticular usage of arthroscopes, the surroundings of joints are composed of cartilage; therefore, to prevent cartilage damage, arthroscopes are not usually designed to come into contact with surrounding tissues. When used in the spinal region, the need arises to hold and suspend the scope to secure the field of view. As a result, it becomes necessary to first treat the muscle layer, and there is concern that more damage could occur to the soft tissue layer. In addition, visualization will be unstable due to the shaking of the camera. On the other hand, the FESS scope can be used within the confines of a sleeve, and it is possible to stably hold the camera by applying the beveled part of the sleeve to the bone. Moreover, tissue damage may potentially be reduced as the sleeve can be directly applied to the bone to ablate the soft tissues. From the above, we believe that the use of the FESS scopes is a useful option for spine surgery to secure a stable field of view. In addition, as shown in Figure 3A, it is possible to hold the scope head by placing one side of the sleeve against the bone and the other side around the surgeon's chest, freeing up both hands. This technique is not possible with a standard arthroscope. If the same technique were to be performed arthroscopically, the sleeve would need to be reconstructed.

Fluid irrigation

Continuous fluid irrigation is essential to maintain a clear surgical field. The UBE technique using an arthroscope often requires enlarging the working portals to ensure irrigation. The FESS scope can be combined with a sleeve that allows the insertion of surgical instruments while providing continuous fluid irrigation. Therefore, there is no concern regarding fluids being discharged from the working portal. In addition, the skin incision is also small.

Hemostasis

Plasma-based radiofrequency (PRF), commonly used in UBE, conducts current from the tip of the coagulator to the dorsal side [5]. Plasma is created on the surface, and current flows to the dorsal side. Therefore, when stopping bleeding in the epidural venous layer, there is a concern that electricity may be applied around the nerve. On the other hand, HFB is designed so that electricity flows at the tip alone; therefore, it is possible to conduct electricity locally to enable hemostasis in an isolated area. In addition, the device can be used like a Penfield dissector to create a clear visualization of bony edges. We believe the accuracy and isolation that HFB can provide in bipolar tissue hemostasis is useful in the spinal region, which has bones, soft tissue, and nerves. In this report, we were able to complete the surgery using only HFB. Facilities that perform FESS are likely to already have HFB equipment; thus, this function can be obtained without new investment.

Compared to scopes used in arthroscopy, the diameter of the 6.9 mm scope used in this study is larger. Therefore, when performing treatment on the contralateral side via the subspinous process, decompression is required to secure the visual field. Considering the possibility that more decompression may be required than is necessary for treatment, a narrower scope may be more useful in cases that necessitate a low-profile scope.

## Conclusions

In this technical report, we described the use of FESS scope and HFB in a patient with central stenosis. We were able to obtain a stable visual field, provide bipolar tissue hemostasis in an isolated area around the nerves, and minimize the skin incision by improving fluid irrigation. The UBE technique using an arthroscopic device is considered to be an extension of FESS surgery, and assisted full-endoscopic spine surgery (AFESS) will further develop in combination with FESS technology in the spinal region.
